# Utilizing a Combination of Network Pharmacology and Experimental Validation to Unravel the Mechanism by Which Kuanxiongzhuyu Decoction Ameliorates Myocardial Infarction Damage

**DOI:** 10.3390/medicina59101740

**Published:** 2023-09-28

**Authors:** Yihao Wu, Miaofu Li, Liuying Chen, Linhao Xu, Yizhou Xu, Yigang Zhong

**Affiliations:** 1 Department of Cardiology, Affiliated Hangzhou First People’s Hospital, Zhejiang University School of Medicine, Hangzhou 310006, China; wyh881009@163.com (Y.W.); 12118400@zju.edu.cn (M.L.); lychen93@163.com (L.C.); xulinhaoluck@163.com (L.X.); 2 Translational Medicine Research Center, Affiliated Hangzhou First People’s Hospital, Zhejiang University School of Medicine, Hangzhou 310006, China

**Keywords:** Kuanxiongzhuyu decoction, post-MI, network pharmacology

## Abstract

*Background and Objectives*: With the growing incidence and disability associated with myocardial infarction (MI), there is an increasing focus on cardiac rehabilitation post-MI. Kuanxiongzhuyu decoction (KXZY), a traditional Chinese herbal formula, has been used in the rehabilitation of patients after MI. However, the chemical composition, protective effects, and underlying mechanism of KXZY remain unclear. *Materials and Methods*: In this study, the compounds in KXZY were identified using a high-performance liquid chromatography-mass spectrometry (HPLC-MS) analytical method. Based on the compounds identified in the KXZY, we predictively selected the potential targets of MI and then constructed a protein–protein interaction (PPI) network to identify the key targets. Furthermore, the DAVID database was used for the GO and KEGG analyses, and molecular docking was used to verify the key targets. Finally, the cardioprotective effects and mechanism of KXZY were investigated in post-MI mice. *Results*: A total of 193 chemical compounds of KXZY were identified by HPLC-MS. In total, 228 potential targets were obtained by the prediction analysis. The functional enrichment studies and PPI network showed that the targets were largely associated with AKT-pathway-related apoptosis. The molecular docking verified that isoguanosine and adenosine exhibited excellent binding to the AKT. In vivo, KXZY significantly alleviated cardiac dysfunction and suppressed AKT phosphorylation. Furthermore, KXZY significantly increased the expression of the antiapoptotic proteins Bcl-2 and Bcl-xl and decreased the expression of the proapoptotic protein BAD. *Conclusions*: In conclusion, the network pharmacological and experimental evidence suggests that KXZY manifests anti-cardiac dysfunction behavior by alleviating cardiomyocyte apoptosis via the AKT pathway in MI and, thus, holds promising therapeutic potential.

## 1. Background

Myocardial infarction (MI) is currently a major cause of morbidity and mortality worldwide, posing a significant public health challenge with enormous medical and societal consequences [1]. While thrombolysis, percutaneous coronary intervention, and coronary-artery-bypass grafts remain the most commonly used and effective treatments for MI, they are unable to prevent the irreversible damage to myocardial cells and the series of repair and wound-healing responses following MI. Excessive and extended remodeling of the left ventricle can often result in further deterioration of cardiac function and ultimately lead to heart failure, with a 5-year mortality rate approaching 50% [2]. Consequently, new and validated treatment agents or targets are urgently needed to alleviate the cardiomyocyte injury, prevent the deterioration of irreversible myocardial function, and improve prognoses for patients post-MI.

The use of natural and herbal remedies to treat human problems has been well known for a significant length of time. Because of its holistic and systematic nature, traditional Chinese medicine (TCM) has recently attracted global attention [3]. Thus, herbal remedies are valuable in the search for novel agents to treat myocardial damage post-MI. In the clinic, post-MI patients are mainly associated with the Qi-deficiency-and-blood-stasis syndrome in TCM. This Qi-deficiency-and-blood-stasis syndrome is one of the most common syndromes in TCM, characterized by chest distress, shortness of breath, heart palpitations, tiredness, rapid pulse, etc. Its pathogenic process resembles that of restricted blood flow and cardiac dysfunction, as revealed by modern science [4]. Thus, Qi-supplementation and blood-stimulation therapies are widely used in TCM. In clinical settings, Kuanxiongzhuyu decoction (KXZY), a classical Chinese herbal formula derived from Siwu decoction, showed effectiveness in treating MI patients. As one of the characteristic treatments for the syndrome of Qi deficiency and blood stasis, KXZY can enhance blood regeneration, promote blood circulation, and eliminate blood stasis. Modern medicine has confirmed that several of these herb-extract mixtures have lipid-reduction, anti-inflammatory, immunomodulatory, and antiatherogenic activities [5,6]. In particular, *Conioselinum Chuanxiong* and *Angelica sinensis* (Oliv.) Diels improve myocardial energy metabolism, promote hematopoiesis, and reduce blood deposition [7,8]. Nevertheless, investigations elucidating the protective effect and mechanism of KXZY remain insufficient due to its complex components and the absence of efficient study approaches. Consequently, there is an urgent need to establish an applicable and effective strategy to elucidate complex herbal formulas.

**Figure 1:**
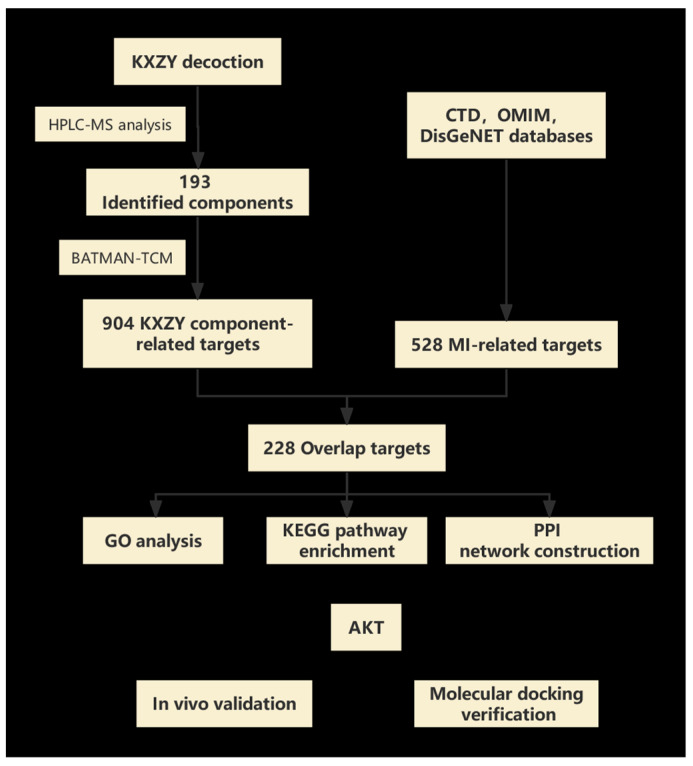
Study flowchart. Flowchart showing the process of this study.

The approaches of network biology and multitarget drug development have made tremendous strides over the past several years. Thus, some new research concepts, such as network pharmacology, have been created, providing an applicable and efficient research strategy for modern drug research and novel therapy discovery [9]. The primary concept of network pharmacology is quite similar to the holistic approach of TCM. It enables researchers to use a holistic view to completely understand the effectiveness and mechanisms of complex herbal formulas. Therefore, network pharmacology is widely utilized in contemporary TCM research. In our study, we integrated HPLC-MS analytical techniques, network pharmacology, and in vitro experiments to determine the chemical composition, potential protective effects, and mechanism of KXZY. We utilized the research strategy of network pharmacology to predict the potential targets based on the results of our HPLC-MS analysis. An MI mouse model was established to evaluate the protective effects of KXZY and to validate the underlying mechanism (Figure 1).

## 2. Methods

### 2.1. Preparation for KXZY

KXZY is composed of sixteen Chinese herbs (Table 1), namely, *Conioselinum Chuanxiong* (Chuanxiong), *Paeonia lactiflora* Pall. (Chishao), *Rehmannia glutinosa* (Gaertn.) DC. (Dihuang), *Angelica sinensis* (Oliv.) Diels (Danggui), *Prunus persica* (L.) Batsch (Taoren), *Carthamus tinctorius* L. (Honghua), *Citrus aurantium* L. (Zhike), *Bupleurum chinense* DC. (Chaihu), *Achyranthes bidentata* Blume (niuxi), *Allium macrostemon* Bunge (Xiebai), *Trichosanthes kirilowii* Maxim. (Gualou), *Ziziphus jujuba* Mill. (Hongzao), *Hirudo nipponica* Whitman. (Shuizhi), *Alpinia katsumadai* Hayata (Doukou), *Platycodon grandiflorus* A.DC. (Jiegeng), and *Glycyrrhiza uralensis* Fisch. ex DC. (Gancao). All of these ingredients were provided by the Hangzhou First People’s Hospital (Hangzhou, China). First, the herbal mixture was soaked in water (1:10, *w*/*v*) for 30 mins and then extracted twice at reflux for 2 times (1 h per cycle). The extracted herbal mixtures were then blended, concentrated, and freeze-dried into a powder for storage.

### 2.2. HPLC-MS Characterization of Primary Chemical Constituents in KXZY

HPLC-MS analysis was conducted on a Q Exactive plus Orbitrap LC-MS/MS (Thermo Fisher, Waltham, MA, USA) equipped with a Waters ACQUITY UPLC HSS T3 column (100 mm 2.1 mm i.d., 1.8 m); the column temperature was 35 °C. The parameter conditions were as follows: injection volume: 10 L; flow rate: 0.3 mL/min; mobile phase: A (deionized water, 0.1% *v/v* formic acid) and B (acetonitrile, 0.1% *v/v* formic acid). Using a gradient program, the following profile was created: 0 min (100% A, 0% B), 10 min (70% A, 30% B), 25 min (60% A, 40% B), 30 min (50% A, 50% B) 0% B, 40 min (30% A, 70% B), 45 min (0% A, 100% B), 60 min (0% A, 100% B), and 70 min (100% A, 0% B). Q Exactive Orbitrap high-resolution mass spectrometry was used for mass spectrometry (MS) data acquisition, and the detection mode was Full MS-ddMS2 with separate scanning in positive and negative ion modes. The parameter conditions were as follows: mass range: 100–1200 m/z; MS1 resolution: 70,000; MS2 resolution: 17,500; ion source voltage: 3.2 kV; capillary temp: 320 °C; aux gas heater temp: 350 °C; sheath gas flow rate: 40 L/min; aux gas flow rate: 15 L/min.

Compound Discover 3.2 software was utilized to extract the characteristic peaks from the raw mass spectrometry data. The mass deviation for elemental matching, molecular formula prediction, and isotopic distribution matching was set to within 5 ppm. To identify the characteristic peaks, two natural product databases, mzcloud and mzVault, were employed. To consider a result positive, it had to meet the following criteria: a mass deviation of less than 5 ppm, isotope distribution matching, and a database match score greater than 70 from the mzVault best match database. All positive results were verified manually, and duplicate results were eliminated.

### 2.3. Prediction and Screening of Targets

Based on the aforementioned prototype components, the possible targets were predicted by a bioinformatics research tool called TCM (BATMAN-TCM) [10]. The predicted targets were rated based on the interactions between possible targets and their likeness to existing targets; those with scores >20 were chosen.

MI-associated targets were compiled by merging the Comparative Toxicogenomics (CTD), Online Mendelian Inheritance in Man (OMIM), and DisGeNET databases [11,12], and “*Homo sapiens*” was the only species considered.

Potential KXZY targets for treating MI were the targets shared by KXZY component-related targets and MI-related targets.

### 2.4. Protein–Protein Interaction (PPI) Network Construction

To examine the probable interactions between target-related genes, we imported these genes into the public STRING database [13]. STRING is often applied to collect comprehensive information on protein interactions between genes, and targets with interaction scores of 0.400 were chosen. Afterward, we loaded all of the above results into Cytoscape to display the connections and screened the key genes using plugins. The Cytoscape plugin CytoNCA was used to filter hub genes by combining several centrality measure calculations, analyses, and assessments. “Betweenness centrality” (BC), “closeness centrality” (CC), and “degree centrality” (DC) are the topological parameters.

### 2.5. Gene and KEGG Analysis

We used the DAVID V6.8 database to perform GO and KEGG pathway enrichment analyses to show the roles of targets for the active components of TCM in terms of gene function and particular signaling pathways [14]. GO analysis was then utilized to annotate gene function via the use of three modules: biological process (BP), molecular function (MF), and cellular component (CC). The cutoff threshold for statistical significance in the DAVID analysis was a *p* value of less than 0.05.

### 2.6. Molecular Docking

AutoDockTools 1.5.6 was used to accomplish molecular docking. The critical target’s atomic coordinates were collected from the Protein Data Bank (PDB) and generated in AutoDockTools-1.5.6 by eliminating water molecules, adding charge, and parameterizing. Downloaded from the TCMSP Database, the 3D structures of active components were built in AutoDockTools by calculating atomic partial charges and parameterizing. The docking site was positioned in the middle of the original ligand inside a cuboid box, and a grid map of each atom type within the box was calculated. Molecular docking of possible targets and components was simulated using the AutoDockTools-1.5.6 program. Each compound’s highest-scoring conformer was examined and displayed using AutoDockTools-1.5.6 and PyMOL.

### 2.7. Animal Model and Groups

Zhejiang Chinese Medical University (Animal Care and Use Committee) approved the procedures for this investigation (Approval No. IACUC-20211101-07). Shanghai SLAC Laboratory Animal Co. provided male C57/BL6 mice weighing between 22 and 24 g at the outset (Shanghai, China). Prior to surgical modeling, experimental mice were acclimated to the laboratory setting for one week. Following surgery, mice were randomly divided into four groups (five to eight mice per group): the sham group (sham), the MI model group (MI), the KXZY group (KXZY, 3.7 g/kg/d), and the captopril group (Cap, 30 mg/kg/d). The medication dosage was determined using the body surface area. As mentioned earlier, surgery was performed in the Model and KXZY groups by ligating the left anterior descending (LAD) coronary artery. Without ligating the LAD coronary artery, a suture was passed across the LAD of the sham group. All groups received intragastric delivery of water or medication for 28 days. Four weeks after an acute myocardial infarction, the heart tissues of sacrificed mice were swiftly removed. A part of the heart was preserved in 4% paraformaldehyde, and another part was kept at −80 °C for future research.

### 2.8. Echocardiography

The supine posture was maintained after intraperitoneal injections of 1% pentobarbital sodium rendered the experimental mice unconscious. The left ventricular function in mice was evaluated by transthoracic echocardiography (Vevo TM 2100, VisualSonics, Toronto, ON, Canada). Under the parasternal short-axis (SAX) ultrasound section, we captured the LVIDd and LVIDs. After this, the EF and FS were separately assessed.

### 2.9. NT-proBNP in Serum

The abdominal aorta of mice was used to collect whole blood, which was then processed to isolate serum and stored at −80 °C for further research. The levels of NT-proBNP in serum were measured using a commercial ELISA kit (Elabscience Biotechnology Co., Ltd., Wuhan, China).

### 2.10. Histopathological

Cardiac tissues treated with paraformaldehyde were embedded in paraffin and cut into 4 μm thick slices. Subsequently, the sections were processed by hematoxylin-eosin (H&E) and Sirius red staining to evaluate the extent of fibrosis and pathological changes. The reagents were purchased from Abcam (Cambridge, MA, USA).

### 2.11. Western Blotting

Total protein was extracted from cardiac tissues using radioimmunoprecipitation assay (RIPA) buffer, including a protease inhibitor cocktail. A 12% SDS polyacrylamide gel electrophoresis (SDS-PAGE) was used to separate the proteins. Then, the proteins were transferred to polyvinylidene fluoride (PVDF) membranes (Millipore, Burlington, MA, USA), which were incubated overnight at 4 °C. Primary antibodies against BAD, AKT, and p-AKT were obtained from Cell Signaling Technology (Danvers, MA, USA), while Bcl-2, Bcl-xl, and GAPDH were purchased from Abcam (Cambridge, MA, USA). Then, secondary antibodies (Abcam, Cambridge, MA, USA) were incubated at 25 °C for 1 h. Bands were visualized using enhanced chemiluminescence (ECL) plus Western blotting detection reagents (Bio-Rad, Hercules, CA, USA) and subsequently captured by the ChemiDoc Touch Imaging System and Image Lab software (Bio-Rad, Hercules, CA, USA).

### 2.12. Statistical Analysis

All animal experiment results are reported as the mean ± SD. SPSS 17.0 statistical software was used for the statistical analysis (IBM, Armonk, NY, USA). For numerous comparisons across groups, one-way analysis of variance (ANOVA) was applied in line with tests for normality and homogeneity of variance. *p* values less than 0.05 were considered statistically significant.

## 3. Results

### 3.1. Characterization of the Chemical Constituents in KXZY

**Figure 2:**
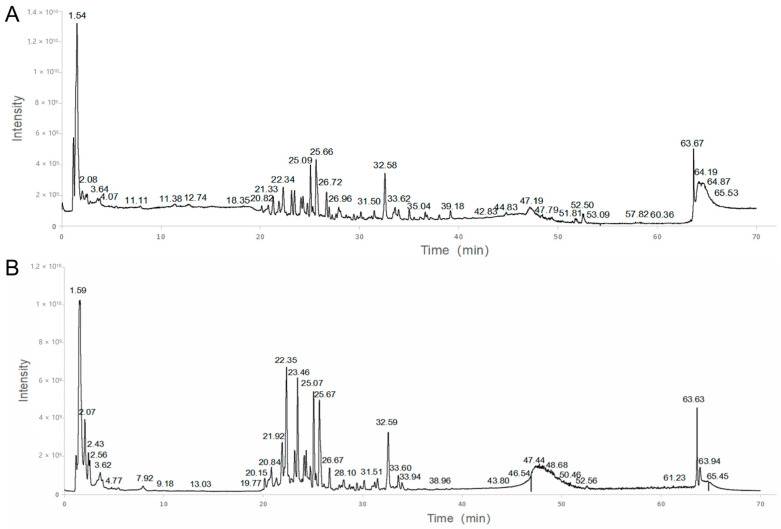
Total ion chromatogram (TIC) in positive mode (**A**) and negative mode (**B**) of KXZY.

**Figure 3:**
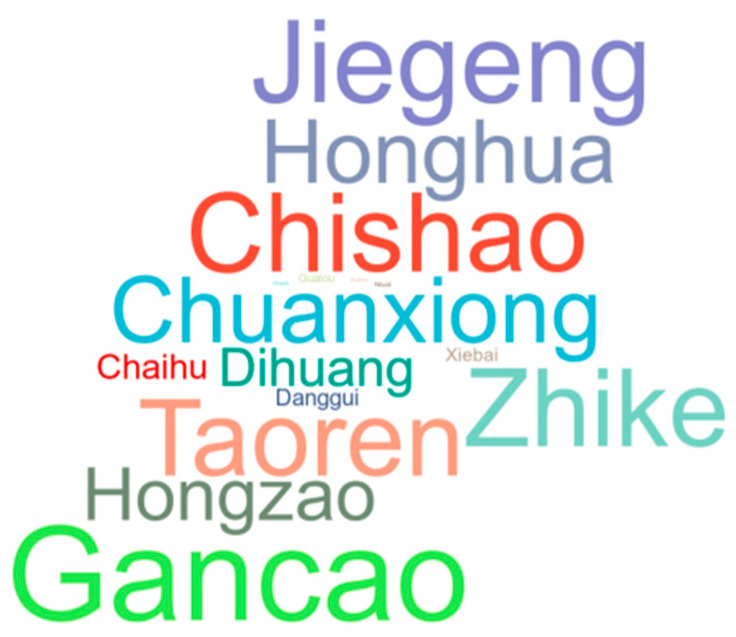
Relative content percentages of each herb. Different colors indicate different herbs, and font size indicates the content.

Figure 2 depicts the representative chromatogram acquired by HPLC-MS analysis. Based on the HPLC-MS results, we matched the previous research and databases, and a total of 193 compounds (Table 2) were identified [15,16,17,18,19]. The abundance of the compounds was inferred from the relative peak areas, and the compounds with high relative contents included amygdalin, naringin, paeoniflorin, neohesperidin, diammonium glycyrrhizinate, betaine, sucrose, stachydrine, safflomin A, 18 β-glycyrrhetintic acid, 2-pyrrolidinecarboxylic acid, citric acid, and naringenin. In addition, we searched the herb sources of the compounds and summed the abundance of compounds attributed to each herb to calculate the relative content percentages. Among them, the highest content of compounds was found for Gancao, followed by Jiegeng and Chishao, as shown in Figure 3.

### 3.2. Screening of the Potential Targets of KXZY in Treating MI

Then, the above compounds were input into BATMAN to predict the related targets, and targets with scores > 20 were considered meaningful. After eliminating duplicated genes, a total of 90 vital components with 904 targets were retrieved.

**Figure 4:**
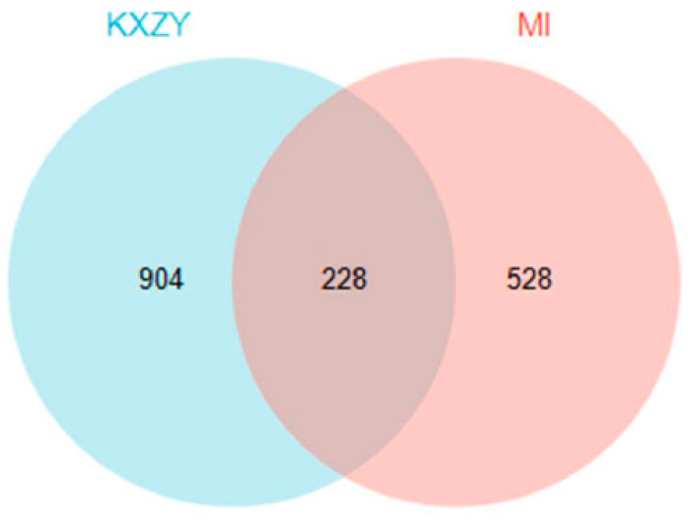
The common targets between KXZY and MI. The blue circles indicate KXZY targets, and the pink circles indicate MI targets.

In total, 757 genes were identified from the CTD, OMIM, and DisGeNET databases that met the requirements for a connection with MI. After eliminating duplicated genes, 528 MI-related genes were retrieved. As shown in Figure 4, the screened drug and disease targets were intersected to obtain 228 common targets between KXZY and MI, They were used as the potential targets of KXZY in treating MI in subsequent network construction and pathway enrichment analysis.

### 3.3. GO and KEGG Analysis

**Figure 5:**
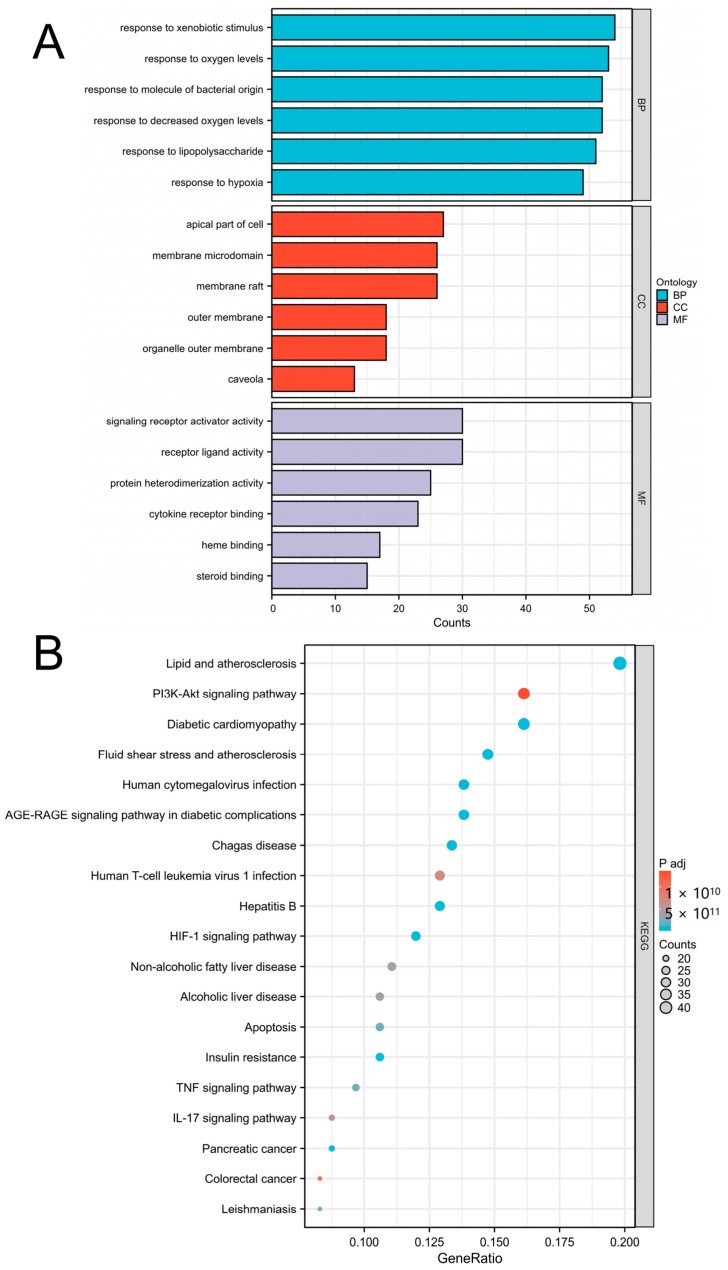
Major enrichment analyses of GO terms and KEGG pathways. (**A**) GO term analysis included BP (blue), CC (red), and MF (gray). (**B**) KEGG pathways. The area of the dot indicates the number of enriched genes; the larger the dot is, the greater the number of genes. The color of the dot represents the significance of the *p* value; the pathway with an intense red color indicates a significant *p* value.

As Figure 5 shows, GO analysis revealed that the following terms were significantly enriched: For BP, the top 3 terms were response to decreased oxygen levels, response to oxygen levels, and response to hypoxia. For CC, the top 3 terms were membrane raft, membrane microdomain, and apical part of cell. For MF, the top 3 terms were receptor ligand activity, signaling receptor activator activity, and protein heterodimerization activity. KEGG enrichment results indicated that these targets were mostly enriched in lipid and atherosclerosis, the PI3K-Akt signaling pathway, the cAMP signaling pathway, and the HIF-1 signaling pathway.

### 3.4. PPI Network Analysis

**Figure 6:**
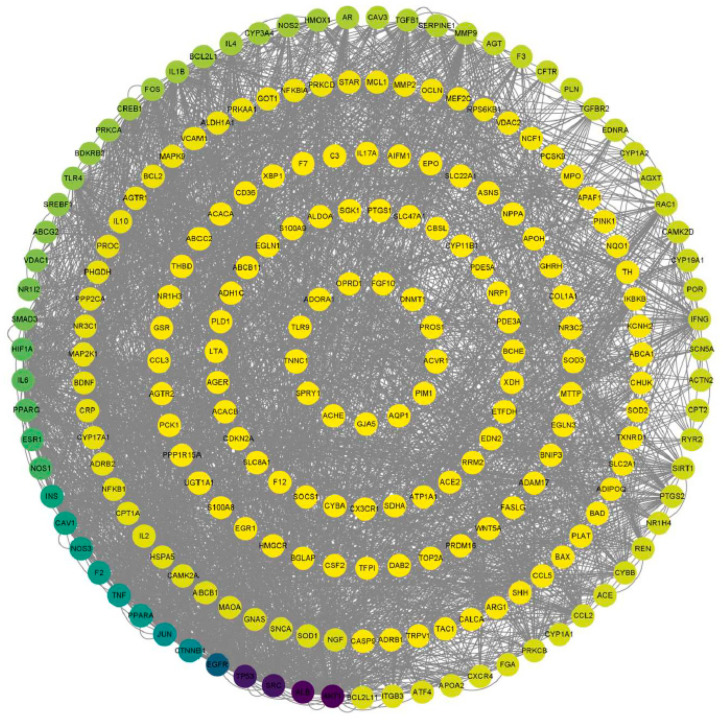
Topological analysis of the PPI network. Nodes represent target proteins (the color of the dot represents the significance of BC, and the dot with an intense black color indicates a significant BC). Edge represents protein–protein association.

To analyze potential protein interactions, the predicted targets of KXZY were uploaded into the SPRING database. As shown in Figure 6, a PPI network was constructed involving 228 nodes and 1327 edges. CytoNCA, a plug-in for Cytoscape, was applied to conduct topological analysis. According to the outcomes of the CytoNCA analysis, there were 62 key nodes whose betweenness centrality (BC) was greater than the mean (betweenness centrality = 379.2568782); this represented 28.44% of the total number of nodes. The top 10 hub genes were as follows: AKT1, ALB, SRC, TP53, EGFR, CTNNB1, JUN, PPARA, TNF, and F2. The complete results are illustrated in Table 3. Our findings revealed that these genes play an important role as network hubs.

**Figure 7:**
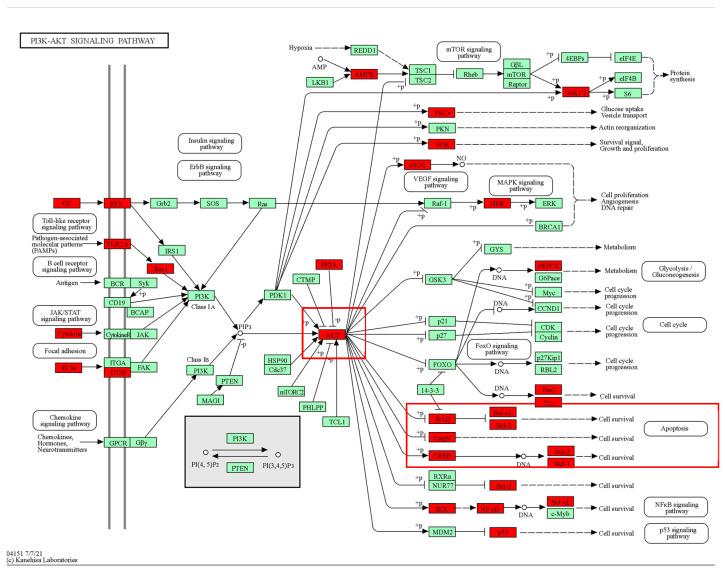
Mapping of the top targets in the PI3K-AKT signaling pathway. Red markers indicate predicted targets. The red box indicates that hub targets are enriched in the AKT/Bcl-2/Bax-associated apoptosis pathway.

KEGG pathway enrichment results indicated that there was significant enrichment in the PI3K/AKT signaling pathway. AKT1 was the top hub target in the structural network. Therefore, we inferred that the PI3K/AKT signaling pathway plays a critical role after MI. Thus, we further mapped the top-ranked hub targets in the PI3K/AKT pathway. As shown in Figure 7, a significant proportion of hub targets were associated with the PI3K/AKT pathway. Notably, apoptosis regulation-related genes, including BAD, Bcl-2, and Bcl-xl, were significantly labeled. Based on the above results, we inferred that KXZY might exert anti-apoptotic effects by regulating the AKT protein. The top hub gene was AKT1.

### 3.5. Molecular Docking Verification

**Figure 8:**
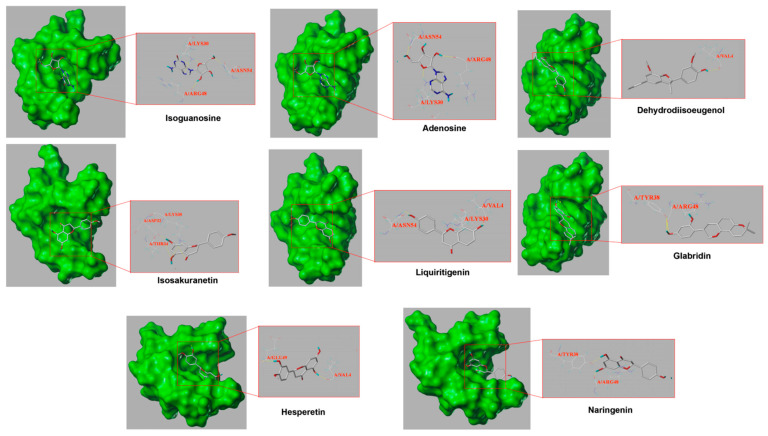
Part of the molecular docking results. The left half represents the atom binding diagram of the molecule and protein, and the right half represents the enlarged picture. The AKT1 structure is presented as a green sphere, the skinny stick represents the residue, and the compound structure is presented as a thick stick (gray represents carbon atoms, red oxygen atoms, blue nitrogen atoms, and light blue hydrogen atoms, and the dotted yellow line represents the interactions).

Finally, we screened the key active compounds that precisely hit AKT1, which is the key target protein, based on the above target prediction results (isoguanosine, naringenin, liquiritigenin, isosakuranetin, hesperetin, dehydrodiisoeugenol, glabridin, and adenosine). As shown in Table 4, isoguanosine and adenosine were the most effective combinations with AKT1. However, adenosine was relatively low in the HPLC analysis. The PyMOL results are visualized in Figure 8.

### 3.6. KXZY Ameliorated Cardiac Dysfunction in Post-MI Mice

**Figure 9:**
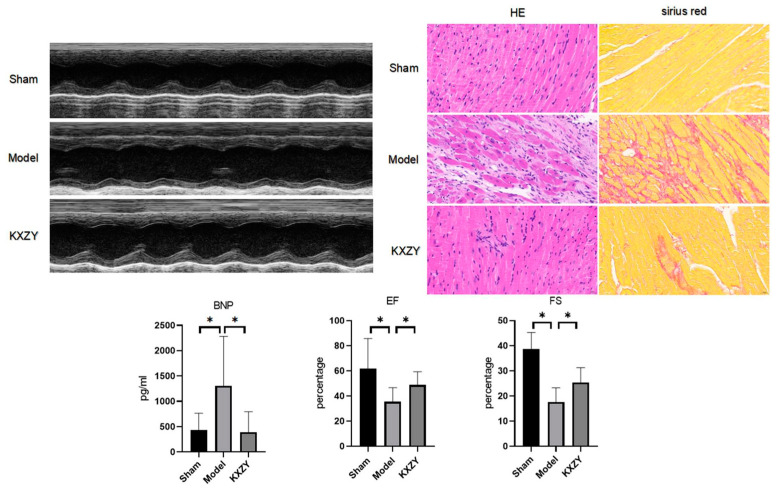
The effects of KXZY on cardiac function and histology in post-MI mice. n = 3; ×40, scale bars, 50 μM; * *p* < 0.05.

To confirm the cardioprotective benefits of KXZY after MI, KXZY was intragastrically administered to post-MI mice for 4 weeks. Subsequently, we evaluated the function and histopathology of the LV. As shown in Figure 9, echocardiographic data revealed that the EF and FS values in the MI group were significantly lower than those in the sham group (*p* < 0.05). Conversely, in mice treated with KXZY or captopril, the EF and FS levels were markedly increased compared with those in the MI group (*p* < 0.05). The results demonstrated that KXZY could improve cardiac function after MI. In addition, our study indicated that the serum levels of NT-proBNP, which is a cardiac dysfunction marker, were markedly elevated in the model group (*p* < 0.05). In contrast, KXZY appreciably attenuated the elevation of NT-proBNP levels (*p* < 0.05).

Moreover, the histopathological results of cardiac tissue revealed disorganized cardiac muscle fiber arrangements, disruption of cardiac structure, and significant interstitial edema in the MI group. The total cardiac fibrosis area was markedly enlarged in the MI group versus the control group. In the KXZY group, these histopathologic abnormalities were restored. These results provide vital evidence that KXZY can attenuate the extent of myocardial injury in post-MI mice.

### 3.7. KXZY Activates the AKT Protein and Attenuates Myocardial Apoptosis

**Figure 10:**
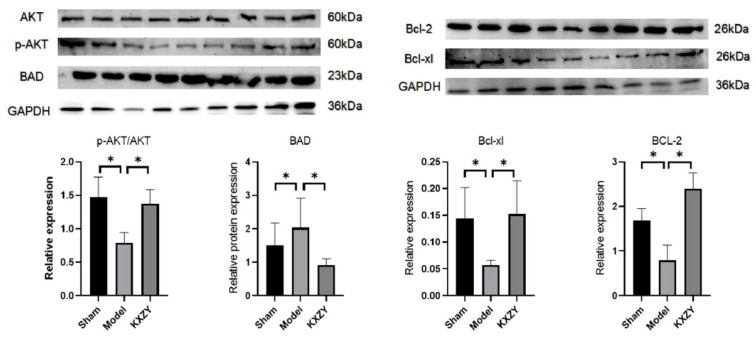
The effects of KXZY on AKT and apoptosis proteins in post-MI mice. n = 3; * *p* < 0.05.

As shown in Figure 10, compared to the sham group, the levels of Bcl-2 and Bcl-xl proteins significantly decreased, while the expression of the BAD protein was significantly increased. Conversely, KXZY increased the expression of Bcl-2 and Bcl-xl proteins and decreased the expression of the BAD protein. Furthermore, KXZY increased the phosphorylation of the AKT protein. When AKT protein becomes activated after phosphorylation, it can inhibit the activity of the proapoptotic protein BAD, thereby leading to an increase in the expression of antiapoptotic proteins such as bcl-2 and bcl-xl. Thus, the results of our study show that FKZF inhibits cardiomyocyte apoptosis by regulating AKT phosphorylation levels and apoptosis-related protein expression.

## 4. Discussion

TCM’s “holistic perspective” and “syndrome distinction” concepts provide unique advantages in the treatment of diseases. As one of the classic prescriptions for promoting blood circulation and removing blood stasis, KXZY has certain effects on the prevention and treatment of cardiovascular diseases. It is usually added according to clinical syndrome differentiation and disease, such as in post-MI patients with chest distress, shortness of breath, heart palpitations, and tiredness. Wang et al.’s research shows that supplementing qi and activating blood therapy were effective and safe in the treatment of coronary heart disease [20]. In addition, during the last decade, tremendous progress has been made in the treatment and study of a variety of illnesses employing a single active component derived from herbs [21,22]. Due to their complicated composition, however, current research into TCM formulas continues to encounter significant obstacles and problems. Due to the rapid growth of bioinformatics and polypharmacy, network pharmacology has been creatively applied in research for TCM formula distinction. In our research, HPLC-MS was used to identify the structural composition of KXZY extracts. Furthermore, we conducted a detailed network pharmacology analysis for target prediction based on the above results. Finally, molecular docking and related experiments were used for validation.

We first identified 193 substances using the HPLC-MS technique. Among these, quercetin, liquiritigenin, and naringin have been reported to have antiapoptotic, antihypertensive, anti-inflammatory, and anti-cardiovascular injury properties [23,24,25]. Then, we constructed the pharmacological network of KXZY based on previous HPLC-MS results. A total of 228 targets were predicted in combination with the MI gene database. GO and KEGG pathway enrichment results indicated that KXZY ameliorated MI damage through multiple biological processes, including response to oxygen levels, response to hypoxia, lipid and atherosclerosis, and regulation of apoptosis. The differential targets were then found to be predominantly enriched in pathways of neurodegeneration—multiple diseases, the PI3K-Akt signaling pathway, the cAMP signaling pathway, and the HIF-1 signaling pathway. Moreover, we screened hub genes using the cytoNCA plug-in and observed that AKT1 was the top target, and the majority of targets were associated with the PI3K-Akt signaling pathway. Specifically, the targets of functional genes were primarily associated with the regulation of apoptosis by the AKT signaling pathway. These findings indicated that AKT-mediated anti-apoptotic alterations may be the crucial target of KXZY.

Cardiomyocyte apoptosis plays an important role during chronic cardiac remodeling and heart failure. Especially in the acute and subacute phases of MI, apoptosis is a prevalent pathogenic characteristic [26]. Studies have shown that the detrimental effect of apoptosis may be more apparent after MI [27]. Moe et al. discovered that the apoptosis of cardiomyocytes occurs over time and correlates strongly with the severity of cardiac dysfunction in heart failure [28]. Baldi et al. found that there was a sustained apoptotic reaction after MI [29]. Wang et al. noted an increase in the number of apoptotic cells 28 days after AMI [30]. Nevertheless, suppression of apoptosis might dramatically increase heart survival and decrease chronic cardiac remodeling and dysfunction [31].

The AKT protein is one of the serine/threonine protein kinase families that regulates several biological activities, including cell growth, metabolism, and survival [32]. The AKT family consists of three isoforms: AKT1, AKT2, and AKT3. AKT1 is the most extensively studied isoform and is widely expressed in a variety of tissues. AKT2 is mainly expressed in skeletal muscle, liver, and adipose tissue. AKT3 is expressed in brain tissue and is involved in regulating neuronal development and function, as well as cell survival and proliferation [33]. The AKT isoform that is mainly expressed in myocardial tissue is AKT1. AKT1 plays a critical role in the regulation of cardiac function, including the control of cardiac hypertrophy, apoptosis (programmed cell death), and contractility. Among them, AKT1 promotes cell survival by inhibiting multiple targets, such as BAD and Bcl-2, in the apoptosis signaling cascade [34]. A growing number of studies have shown that AKT activation prevents cardiomyocyte death, while AKT inhibition exacerbates cardiomyocyte apoptosis and cardiac dysfunction [35]. AKT inactivation was responsible for MI-induced myocardial damage, whereas AKT phosphorylation increased cardiomyocyte survival and prevented MI-induced cardiac dysfunction, according to previous findings.

Therefore, based on the above results, further investigation was conducted to explore the potential anti-apoptotic mechanism of KXZY. To assess the potential cardioprotective and anti-apoptotic effects of KXZY, a post-MI mouse model was utilized in this study. Our study shows that post-MI mice exhibit better EF and FS values with the treatment of KXZY. Our research suggested that KXZY could improve left ventricular systolic function in post-MI mice. Furthermore, we assessed the AKT-related anti-apoptotic effects of KXZY after MI damage. AKT phosphorylation was significantly inhibited, and BAD expression was markedly reduced. There was an increase in the expression of Bcl-2 and Bcl-xl. We therefore infer that KXZY may exert its anti-apoptotic effects by regulating the phosphorylation and activation of AKT1, which in turn inhibits the pro-apoptotic protein BAD, allowing Bcl-2 and bcl-xl to inhibit apoptosis and maintain cell survival. These findings indicated that AKT-related antiapoptotic mechanisms might be the main mechanism of KXZY in post-MI damage.

Finally, molecular docking was used to verify the mode of interaction between compounds of KXZY and AKT1 protein molecules. Eight compounds of KXZY were predicted to bind to AKT1: isoguanosine, naringenin, liquiritigenin, isosakuranetin, hesperetin, dehydrodiisoeugenol, glabridin, and adenosine. Our study indicated that isoguanosine exhibits high binding energy through molecular docking. Thus, we inferred that isoguanosine might improve cardiac dysfunction after MI by inhibiting AKT-related apoptosis.

## 5. Conclusions

This study identified 193 chemical compounds of KXZY by HPLC-MS. In addition, 228 potential targets were predicted, and a PPI network was constructed for further network pharmacology research. The findings of functional enrichment analysis suggest that the apoptotic process is strongly associated with AKT1. Additionally, we used a post-MI mouse model to verify the anti-apoptotic and anti-cardiac dysfunction behaviors of KXZY, and isoguanosine and adenosine were found to be two important pharmacodynamic compounds. Consequently, the herbal method provides a wide repository for the development of novel therapeutic agents for the treatment of post-MI cardiac damage, and KXZY might serve as an important supplementary substance.

## Figures and Tables

**Table 1:** The composition of the KXZY decoction.

Latin Name	Chinese Name	Content (g)	Latin Name	Chinese Name	Content (g)
*Conioselinum Chuanxiong*	Chuanxiong	15	*Prunus persica* (L.) Batsch	Taoren	15
*Paeonia lactiflora* Pall.	Chishao	15	*Carthamus tinctorius* L.	Honghua	10
*Rehmannia glutinosa* (Gaertn.) DC.	Dihuang	15	*Citrus aurantium* L.	Zhike	12
*Angelica sinensis* (Oliv.) Diels	Danggui	12	*Bupleurum chinense* DC.	Chaihu	10
*Achyranthes bidentata* Blume	niuxi	10	*Trichosanthes kirilowii* Maxim.	Gualou	10
*Allium macrostemon* Bunge	Xiebai	10	*Ziziphus jujuba* Mill.	Hongzao	15
*Hirudo nipponica* Whitman.	Shuizhi	5	*Alpinia katsumadai* Hayata	Doukou	5
*Platycodon grandiflorus* A.DC.	Jiegeng	5	*Glycyrrhiza uralensis* Fisch. ex DC.	Gancao	8

**Table 2:** Characterization of chemical constituents in KXZY.

No.	Formula	*m*/*z*	tR (min)	Relative Content (%)	Identity
1	C_20_H_27_NO_11_	457.15851	22.524	9.113	Amygdalin
2	C_27_H_32_O_14_	580.17929	25.259	6.855	Naringin
3	C_23_H_28_O_11_	480.16329	23.642	6.453	Paeoniflorin
4	C_28_H_34_O_15_	610.18975	25.837	6.118	Neohesperidin
5	C_42_H_62_O_16_	822.40393	32.766	5.017	Diammonium glycyrrhizinate
6	C_5_H_11_NO_2_	117.07898	1.692	4.596	Betaine
7	C_12_H_22_O_11_	342.11622	1.754	4.577	Sucrose
8	C_7_H_13_NO_2_	143.09474	1.745	3.164	Stachydrine
9	C_27_H_32_O_16_	612.16911	22.087	3.146	Safflomin A
10	C_30_H_46_O_4_	470.3395	32.769	3.117	18 β-Glycyrrhetintic Acid
11	C_5_H_9_NO_2_	115.06339	1.738	2.576	2-Pyrrolidinecarboxylic acid
12	C_6_H_8_O_7_	192.02709	1.85	2.55	Citric acid
13	C_15_H_12_O_5_	272.06828	25.261	2.538	Naringenin
14	C_16_H_14_O_6_	140.04846	25.822	2.427	Hesperetin
15	C_23_H_28_O_11_	526.16871	23.362	2.405	Albiflorin
16	C_21_H_22_O_9_	418.12653	24.504	2.018	Liquiritin
17	C_15_H_12_O_4_	256.07341	24.51	1.744	Isoliquiritigenin
18	C_21_H_18_O_11_	446.08497	26.856	1.446	Baicalin
19	C_21_H_22_O_8_	402.13164	35.226	1.328	Nobiletin
20	C_20_H_17_NO_4_	335.11565	28.127	1.272	Epiberberine
21	C_16_H_18_O_9_	354.09513	22.341	1.188	Cryptochlorogenic acid
22	C_9_H_11_NO_2_	165.07916	12.97	1.168	L-Phenylalanine
23	C_24_H_42_O_21_	666.22231	2.624	0.943	Stachyose
24	C_7_H_7_NO_2_	137.04779	1.742	0.886	Trigonelline HCl
25	C_12_H_16_O_2_	192.1152	36.831	0.874	Senkyunolide A
26	C_27_H_32_O_14_	580.17937	24.925	0.86	Narirutin
27	C_15_H_22_O_10_	362.12149	8.096	0.834	Catalpol
28	C_10_H_12_O_4_	196.07357	25.953	0.788	Xanthoxyline
29	C_10_H_13_N_5_O_4_	267.09683	11.509	0.754	Adenosine
30	C_7_H_12_O_6_	192.06343	1.739	0.746	Quinic acid
31	C_26_H_30_O_8_	470.19427	34.14	0.714	Limonin
32	C_18_H_32_O_16_	504.16943	2.743	0.678	Manninotriose
33	C_20_H_20_O_7_	372.12099	37.022	0.58	Tangeretin
34	C_15_H_24_O_9_	348.14228	21.05	0.564	Ajugol
35	C_30_H_46_O_5_	486.33467	30.333	0.515	Quillaic acid
36	C_27_H_44_O_7_	526.31422	24.34	0.509	Hydroxyecdysone
37	C_6_H_6_O_3_	126.03174	1.762	0.489	5-Hydroxymethylfurfural
38	C_30_H_32_O_12_	584.18985	29.607	0.479	Benzoylpaeoniflorin
39	C_7_H_6_O_5_	170.0216	8.27	0.436	Gallic acid
40	C_22_H_20_O_11_	460.10069	28.866	0.422	Wogonoside
41	C_22_H_22_O_9_	430.12642	26.899	0.401	Ononin
42	C_10_H_8_O_3_	144.02113	24.981	0.385	7-Methoxycoumarin
43	C_5_H_9_NO_4_	147.05327	1.624	0.354	L-Glutamic acid
44	C_15_H_16_O_4_	260.1049	33.622	0.34	Isomeranzin
45	C_42_H_68_O_13_	780.46643	33.774	0.304	Saikosaponin D
46	C_6_H_14_O_6_	182.07915	1.641	0.294	Mannitol
47	C_21_H_21_NO_4_	351.14708	27.846	0.272	Palmatine
48	C_10_H_12_O_4_	196.07359	23.36	0.265	Cantharidin
49	C_10_H_10_O_4_	194.05791	24.982	0.261	Ferulic acid
50	C_20_H_23_NO_4_	341.16277	23.188	0.252	(+)-Magnoflorine
51	C_21_H_22_O_9_	418.12648	26.829	0.252	Isoliquiritin
52	C_9_H_8_O	132.05754	25.953	0.245	Cinnamaldehyde
53	C_15_H_12_O_4_	256.07353	28.242	0.243	Liquiritigenin
54	C_30_H_46_O_3_	454.3452	33.827	0.225	Wilforlide A
55	C_6_H_6_O_3_	126.0317	8.277	0.212	Pyrogallol
56	C_22_H_20_O_11_	460.10067	28.266	0.208	Oroxylin A-7-O-β-D-glucuronide
57	C_6_H_13_NO_2_	131.09473	5.241	0.203	L-Leucine
58	C_28_H_34_O_14_	594.19521	28.294	0.187	Poncirin
59	C_20_H_20_O_7_	372.12102	33.602	0.187	Sinensetin
60	C_42_H_66_O_14_	794.44569	32.631	0.184	Chikusetsu saponin IVa
61	C_30_H_44_O_4_	468.32408	31.685	0.184	Glabrolide
62	C_10_H_13_N_5_O_5_	283.09178	14.099	0.18	Isoguanosine
63	C_27_H_30_O_15_	594.1589	24.846	0.177	Kaempferol-3-O-rutinoside
64	C_27_H_30_O_14_	578.16362	25.254	0.173	Rhoifolin
65	C_57_H_92_O_28_	1224.57672	28.035	0.161	Platycodin D
66	C_27_H_30_O_15_	594.1589	22.692	0.156	Vicenin II
67	C_12_H_14_O_2_	190.09939	32.818	0.153	Ligustilide
68	C_30_H_52_O_26_	828.275	1.778	0.152	Maltopentaose
69	C_15_H_10_O_5_	270.05236	26.861	0.149	Baicalein
70	C_15_H_12_O_6_	288.06331	24.278	0.144	Eriodictyol
71	C_16_H_12_O_4_	268.07334	32.693	0.142	Formononetin
72	C_25_H_24_O_12_	516.12675	25.335	0.142	1,3-Dicaffeoylquinic acid
73	C_25_H_24_O_12_	516.12676	25.787	0.134	Isochlorogenic acid C
74	C_12_H_12_O_2_	188.08374	32.605	0.131	3-Butylidenephthalide
75	C_9_H_16_O_4_	188.105	26.189	0.131	Azelaic acid
76	C_16_H_14_O_5_	286.08406	28.285	0.13	Isosakuranetin
77	C_19_H_18_O_6_	342.11037	33.683	0.126	6-Demethoxytangeretin
78	C_9_H_8_O_3_	164.04756	5.706	0.114	p-Coumaric acid
79	C_22_H_23_NO_4_	365.1628	28.557	0.114	Dehydrocorydaline
80	C_23_H_28_O_12_	382.1653	22.165	0.114	Oxypaeoniflorin
81	C_26_H_28_O_14_	564.14811	23.27	0.111	Vicenin III
82	C_20_H_20_O_7_	372.12108	32.049	0.109	Isosinensetin
83	C_15_H_12_O_5_	272.06846	30.387	0.102	Naringenin chalcone
84	C_29_H_36_O_15_	624.20546	24.312	0.1	Verbascoside
85	C_16_H_12_O_5_	284.06848	34.93	0.099	Wogonin
86	C_42_H_60_O_16_	822.40294	33.795	0.098	Dipotassium glycyrrhizinate
87	C_27_H_30_O_16_	610.15368	24.106	0.097	Rutin
88	C_20_H_19_NO_4_	337.13146	26.237	0.095	Jatrorrhizine
89	C_7_H_6_O_3_	138.03174	26.741	0.095	4-Hydroxybenzoic acid
90	C_16_H_24_O_10_	376.1371	21.383	0.095	Loganic acid
91	C_7_H_6_O_3_	138.03172	21.807	0.091	Protocatechualdehyde
92	C_18_H_32_O_16_	550.175	3.46	0.089	Raffinose
93	C_19_H_15_N_3_O	301.12157	26.661	0.088	Dehydroevodiamine
94	C_16_H_12_O_5_	284.06854	28.64	0.088	Calycosin
95	C_21_H_20_O_11_	448.10066	25.412	0.084	Quercitrin
96	C_25_H_24_O_12_	516.12678	24.963	0.082	Isochlorogenic acid B
97	C_27_H_43_NO_3_	429.32436	26.086	0.08	Peiminine
98	C_26_H_30_O_7_	454.19936	37.521	0.076	Obacunone
99	C_9_H_12_N_2_O_6_	244.06961	6.015	0.075	Uridine
100	C_11_H_12_N_2_O_2_	204.08999	21.475	0.074	L-Tryptophan L
101	C_21_H_20_O_12_	464.09576	24.538	0.073	Hyperoside
102	C_52_H_84_O_24_	1092.53526	27.843	0.068	Desapioplatycodin D
103	C_16_H_14_O_5_	264.10208	27.739	0.066	Licochalcone B
104	C_28_H_32_O_15_	608.17464	25.652	0.064	Diosmin
105	C_48_H_78_O_17_	926.52433	31.227	0.06	Saikosaponin C
106	C_45_H_76_O_19_	920.49865	26.796	0.057	Timosaponin B II
107	C_10_H_10_O_3_	178.06295	23.362	0.054	Ferulaldehyde
108	C_9_H_6_O_3_	162.03176	31.867	0.052	7-Hydroxycoumarin
109	C_9_H_11_NO_3_	164.04756	1.93	0.052	L-Tyrosine
110	C_16_H_22_O_10_	374.12135	21.063	0.051	Geniposidic acid
111	C_21_H_20_O_6_	368.12612	35.986	0.05	Icaritin
112	C_15_H_10_O_6_	286.04769	24.836	0.046	Kaempferol
113	C_21_H_34_O_14_	510.1951	20.891	0.042	Rehmannioside C
114	C_12_H_14_O_2_	190.09938	37.346	0.04	3-n-Butylphathlide
115	C_15_H_10_O_4_	254.05791	27.861	0.04	Daidzein
116	C_20_H_20_O_8_	388.11583	32.396	0.038	5-O-Demethylnobiletin
117	C_16_H_14_O_4_	270.08924	30.378	0.037	Retrochalcone
118	C_9_H_6_O_4_	178.0267	23.198	0.036	5,7-Dihydroxychromone
119	C_16_H_22_O_9_	404.13197	23.024	0.033	Sweroside
120	C_21_H_18_O_13_	478.07471	24.543	0.032	Quercetin 3-O-β-D-Glucuronide
121	C_9_H_8_O_2_	148.05246	24.979	0.032	Cinnamic acid
122	C_42_H_62_O_16_	822.40337	34.38	0.032	Glycyrrhizic acid
123	C_9_H_6_O_4_	178.0267	22.957	0.031	Esculetin
124	C_27_H_45_NO_3_	431.34023	27.45	0.031	Peimine
125	C_22_H_33_NO_4_	375.24114	25.149	0.031	Tuberostemonine
126	C_39_H_64_O_13_	740.4348	26.791	0.03	Timosaponin A-III
127	C_15_H_10_O_6_	286.04784	28.573	0.029	Luteolin
128	C_19_H_21_NO_2_	295.1573	27.028	0.029	Nuciferine
129	C_17_H_24_O_11_	404.13197	23.398	0.028	Secoxyloganin
130	C_16_H_20_O_9_	356.11076	22.001	0.028	Gentiopicrin
131	C_21_H_20_O_11_	448.10059	24.572	0.028	Cynaroside
132	C_22_H_20_O_12_	476.09565	28.42	0.027	Scutellarin methyl ester
133	C_20_H_28_O_8_	442.18434	26.17	0.027	Lobetyolin
134	C_5_H_5_N_5_	135.05459	11.536	0.026	Adenine
135	C_21_H_20_O_10_	450.1163	24.284	0.026	Vitexin
136	C_18_H_18_O_2_	266.13081	41.291	0.026	Magnolol
137	C_15_H_14_O_6_	290.07891	22.367	0.025	Epicatechin
138	C_16_H_12_O_5_	284.06852	35.699	0.025	Oroxylin A
139	C_20_H_22_O_4_	294.12567	26.507	0.025	Dehydrodiisoeugenol
140	C_15_H_10_O_5_	270.05266	30.521	0.025	Genistein
141	C_18_H_19_NO_4_	313.13127	22.768	0.025	Norisoboldine
142	C_16_H_12_O_6_	300.06334	28.226	0.025	Tectorigenin
143	C_15_H_22_O_9_	346.12661	20.597	0.024	Aucubin
144	C_11_H_10_O_4_	206.05811	27.335	0.024	Scoparone
145	C_35_H_46_O_20_	786.25868	22.772	0.024	Purpureaside C
146	C_21_H_32_O_15_	570.17995	6.237	0.024	Rehmannioside A
147	C_8_H_8_O_4_	168.0422	21.029	0.023	4-Methoxysalicylic acid
148	C_20_H_18_O_8_	386.10022	34.413	0.022	Irisflorentin
149	C_16_H_12_O_7_	316.05809	24.915	0.022	Isorhamnetin
150	C_20_H_19_NO_5_	353.1263	25.591	0.022	Hydroprotopine
151	C_19_H_18_O_7_	358.10536	35.719	0.022	Gardenin B
152	C_16_H_12_O_5_	284.06855	28.26	0.021	Glycitein
153	C_24_H_42_O_21_	712.2278	5.877	0.021	Nystose
154	C_22_H_22_O_10_	446.12151	24.231	0.019	Calycosin-7-O-β-D-glucoside
155	C_27_H_41_NO_3_	427.30892	25.186	0.019	Peimisine
156	C_15_H_10_O_7_	302.04253	24.115	0.019	Quercetin
157	C_17_H_14_O_7_	330.07405	33.267	0.019	Aurantio-obtusin
158	C_15_H_18_O_2_	230.13088	36.582	0.018	Dehydrocostus lactone
159	C_12_H_8_O_4_	234.05279	32.869	0.018	Isobergapten
160	C_21_H_20_O_9_	416.11067	23.918	0.018	Daidzin
161	C_21_H_32_O_15_	524.17443	20.594	0.018	Melittoside
162	C_18_H_13_N_3_O	287.10588	37.293	0.018	Rutaecarpine
163	C_9_H_6_O_2_	146.03679	23.242	0.018	Coumarin
164	C_24_H_28_O_4_	380.19884	45.529	0.018	Levistilide A
165	C_17_H_26_O_10_	436.15822	22.743	0.018	Loganin
166	C_19_H_15_NO_4_	321.10017	36.512	0.017	Berberrubine
167	C_18_H_16_O_8_	360.08458	31.507	0.015	Irigenin
168	C_21_H_20_O_10_	432.10584	25.616	0.015	Apigenin-7-O-β-D-glucoside
169	C_26_H_30_O_9_	486.18927	33.151	0.014	Rutaevin
170	C_19_H_17_N_3_O	303.13722	36.5	0.014	Evodiamine
171	C_22_H_24_O_10_	224.06841	24.951	0.013	Isosakuranin
172	C_15_H_20_O_2_	232.14639	40.664	0.013	Atractylenolide II
173	C_18_H_16_O_8_	360.08497	26.104	0.012	Rosmarinic acid
174	C_20_H_28_O_10_	428.16835	25.585	0.012	Rosarin
175	C_19_H_17_NO_4_	323.11583	26.413	0.011	Tetrahydrocoptisine
176	C_28_H_34_O_14_	594.19519	27.919	0.011	Didymin
177	C_21_H_20_O_10_	432.1057	28.54	0.011	Emodin-8-O-β-D-glucopyranoside
178	C_21_H_22_O_7_	403.16338	41.635	0.011	Praeruptorin A
179	C_21_H_18_O_12_	462.08028	26.146	0.01	Luteolin 7-glucuronide
180	C_18_H_30_O_2_	278.22466	38.627	0.01	α-Linolenic acid
181	C_21_H_22_O_4_	338.15209	37.984	0.009	Licochalcone A
182	C_15_H_20_O_4_	264.13609	28.098	0.009	Abscisic acid
183	C_13_H_10_O_5_	246.0529	32.952	0.009	Pimpinellin
184	C_16_H_14_O_4_	270.08934	36.737	0.009	Medicarpin
185	C_12_H_8_O_4_	216.04244	31.373	0.008	8-Methoxypsoralen
186	C_47_H_76_O_18_	974.50917	29.657	0.008	Asperosaponin VI
187	C_11_H_6_O_3_	186.03176	30.685	0.008	Isopsoralen
188	C_15_H_12_O_7_	304.05852	25.151	0.008	Taxifolin
189	C_16_H_16_O_6_	304.09474	28.479	0.007	Oxypeucedanin hydrate
190	C_20_H_20_O_4_	324.13641	39.171	0.006	Glabridin
191	C_20_H_18_O_4_	322.12059	35.923	0.005	Neobavaisoflavone
192	C_21_H_20_O_9_	416.11113	25.55	0.005	Puerarin
193	C_31_H_42_O_17_	686.24281	22.974	0.003	Specnuezhenide

**Table 3:** The top 10 targets of the PPI network.

No.	Target	Betweenness Centrality
1	AKT1	4289.0713
2	ALB	4197.868
3	SRC	3918.9045
4	TP53	3871.7322
5	EGFR	2956.3945
6	CTNNB1	2277.677
7	JUN	2211.387
8	PPARA	2030.4283
9	TNF	2015.5178
10	F2	1972.6385

**Table 4:** The docking affinity and interactions of compounds binding to key targets.

Components	Total Score	Crash	Polar
Isoguanosine	5.5657	−1.3847	3.363
Naringenin	3.4565	−1.8875	1.8116
Liquiritigenin	4.6422	−1.018	0.8693
Isosakuranetin	3.96	−0.7319	1.52
Hesperetin	5.1629	−1.702	1.9341
Dehydrodiisoeugenol	5.3929	−1.1121	0.8985
Glabridin	5.1382	−1.3798	1.0598
Adenosine	5.5657	−1.3847	3.3634

## Data Availability

The original data contributing to the findings presented in this study are included in the article. Further inquiries can be addressed to the corresponding authors.
